# Association of *TP53* polymorphic variants *rs1042522* and *rs1642785* with susceptibility and prognosis of acute lymphoblastic leukemia in a Brazilian Amazon population

**DOI:** 10.1186/s12920-026-02371-0

**Published:** 2026-04-14

**Authors:** Glenda Menezes Nogueira, Luca Gabriel Marques Gonçalves, Thaís Lohana Pereira-Ribeiro, Larissa Silva Santos, Fábio Magalhães-Gama, Nilberto Dias Araújo, Adriana Malheiro, Andréa Monteiro Tarragô, Fabíola Silva Alves-Hanna, Allyson Guimarães Costa

**Affiliations:** 1 Programa de Pós-Graduação em Ciências Aplicadas à Hematologia, Universidade do Estado do Amazonas (UEA), Manaus, Brazil; 2 Programa de Pós-Graduação em Imunologia Básica e Aplicada, Instituto de Ciências Biológicas, Universidade Federal do Amazonas (UFAM), Manaus, Brazil; 3 Diretoria de Ensino e Pesquisa, Fundação Hospitalar de Hematologia e Hemoterapia do Amazonas (HEMOAM), Manaus, Brazil; 4 Programa de Pós-graduação em Ciências da Saúde, Instituto René Rachou - Fundação Oswaldo Cruz (FIOCRUZ-Minas), Belo Horizonte, Minas Brazil; 5 Fundação Hospitalar de Hematologia e Hemoterapia do Amazonas (HEMOAM), Av. Constantino Nery 4397, Chapada, Manaus, CEP 69050-001 AM Brasil

**Keywords:** TP53, Genetic polymorphisms, Acute lymphoblastic leukemia, Prognosis, Population genetics, Pediatric cancer

## Abstract

**Background:**

Tumor suppressor genes play a central role in cancer development, and inherited genetic variation may influence both disease susceptibility and clinical outcomes. This study aimed to investigate the frequency and prognostic relevance of *TP53* polymorphic variants in patients with acute lymphoblastic leukemia (ALL) from an admixed population in the Brazilian Amazon.

**Methods:**

A population-based case–control study was conducted including 193 patients diagnosed with ALL and 215 healthy controls. Germline *TP53* polymorphisms *rs1042522* and *rs1642785* were genotyped, and allele and genotype frequencies were compared between groups. Associations with ALL susceptibility, relapse, and mortality were evaluated using multiple genetic models adjusted for age and sex. Combined genotype and haplotype analyses were performed, and overall survival was estimated using Kaplan-Meier curves and log-rank tests.

**Results:**

The CC genotype and the allele C were more frequent among ALL cases than controls. The allele C of *rs1042522* (*p* = 0.021) and *rs1642785* (*p* = 0.022) was associated with increased susceptibility to ALL. In addition, the allele C of *rs1042522* was associated with a higher risk of relapse (*p* = 0.016) and death (*p* = 0.009). Protective effects against ALL were observed under the recessive model for *rs1042522* (*p* = 0.019) and the codominant model for *rs1642785* (*p* = 0.013). Both variants showed protective associations with mortality under the log-additive model. Combined genotype analysis revealed that the CG and GG genotypes of *rs1042522* were associated with a reduced risk of relapse (*p* < 0.001). Overall survival analysis showed reduced survival associated with the CC genotype, whereas improved survival was observed for the heterozygous CG genotype. Haplotype analysis indicated that the GG haplotype was associated with a reduced risk of ALL (*p* = 0.007) and death (*p* = 0.013).

**Conclusions:**

Our findings suggest that germline *TP53* variants *rs1042522* and *rs1642785* modulate susceptibility and clinical outcomes in ALL, supporting their potential role as prognostic biomarkers. This study highlights the importance of population-based genomic investigations in underrepresented populations.

**Supplementary Information:**

The online version contains supplementary material available at 10.1186/s12920-026-02371-0.

## Introduction

Acute lymphoblastic leukemia (ALL) is a lymphoproliferative hematological malignancy that predominantly affects children, with a peak incidence between 2 and 5 years of age [1, 2]. In Brazil, approximately 11,540 cases of leukemia (acute and chronic) are estimated for each three-year period from 2023 to 2025. In the Northern region of the country, leukemia ranks as the sixth most frequent cancer among both men (4.53 per 100,000) and women (3.64 per 100,000), excluding non-melanoma skin cancers [3].

The etiology of ALL remains largely unknown; however, environmental factors such as ionizing radiation, as well as infections and genetic susceptibility, have been implicated in disease development. Although isolated genetic events do not fully explain leukemogenesis, the association of ALL with congenital syndromes, chromosomal translocations, and polymorphic genetic variants underscores the central role of genetic factors in its pathogenesis. Accordingly, genomic studies have increasingly focused on the impact of gene variations in pathways related to inflammation and tumor suppression [4, 5].

A key gene involved in tumor suppression and activated in response to cellular stress signals, such as DNA damage, is tumor protein 53 (*TP53*), which encodes the p53 phosphoprotein. *TP53* plays a central role in safeguarding genomic integrity by regulating the cellular response to damage and preventing malignant transformation. Through its function as a transcription factor, *TP53* controls the expression of multiple genes involved in critical cellular processes, including apoptosis, cell-cycle arrest, senescence, and DNA repair [6].

Given the indispensable role of *TP53*, the presence of single nucleotide variants (SNVs), particularly *rs1042522*, has been reported to partially impair specific p53 functions, such as apoptotic induction, potentially favoring sustained cellular proliferation [7]. In addition, *rs1042522* has been described as a potential risk factor for acute myeloid leukemia (AML) [8]. In chronic lymphocytic leukemia (CLL), the *TP53* variants *rs1042522*, *rs1642785*, and *rs2909430* have been associated with an increased frequency of somatic *TP53* mutations [9].

However, data regarding hematological malignancies such as ALL especially concerning *TP53* polymorphic variants remain limited in Brazil, with a marked scarcity of information from the Amazon region. To address this gap, the present study investigated the frequency and prognostic relevance of the *TP53* variants *rs1042522* and *rs1642785* in patients with ALL from the Brazilian Amazon, supporting their potential role as prognostic biomarkers in this population.

## Materials and methods

### Study design and study population

This case-control study included a convenience sample of 193 patients diagnosed with acute lymphoblastic leukemia (ALL) and treated at Fundação Hospitalar de Hematologia e Hemoterapia do Amazonas (HEMOAM), a regional reference center that diagnoses approximately 40 new ALL cases per year [10]. Patients of all age groups, both sexes, and unrelated individuals were eligible for inclusion. The control group comprised 215 healthy adult individuals (> 18 years) who underwent blood donation or routine screening at HEMOAM. The use of adult controls was intentional, as the inclusion of children could introduce misclassification bias, given that they may still be within the age range at risk for developing ALL. All controls were screened by serological testing for human immunodeficiency virus (HIV), hepatitis C virus (HCV), hepatitis B virus (HBV), human T-lymphotropic virus types 1 and 2 (HTLV-1/2), syphilis, and Chagas disease to ensure their healthy status. In addition, participants were interviewed to assess comorbidities and other risk factors, following the technical guidelines of the Brazilian Ministry of Health. Individuals with known familial relationships (blood relatives) were excluded to ensure the independence of genetic observations. This exclusion criterion was applied within the case group, within the control group, and between cases and controls when such relationships were identified. Relatedness was assessed based on self-reported information obtained during participant recruitment. Patients who underwent bone marrow transplantation during the follow-up period and those diagnosed with other hematological malignancies were also excluded.

### Ethical issues

This study was approved by the Research Ethics Committee of HEMOAM under approval number 3,335,123/2019 (CAAE: 12615918.9.0000.0009), in accordance with the principles of the Declaration of Helsinki and Resolution No. 466/12 of the Brazilian National Health Council, which governs research involving human subjects.

### Biological sample collection and data acquisition

Approximately 2 mL of peripheral blood was collected by venipuncture into vacuum tubes containing EDTA from participants in both groups. Samples were obtained from the Biorepository of Cellular and Molecular Biomarkers in Acute Lymphoblastic Leukemia (BCM-ALL) at HEMOAM. Sociodemographic data (age, sex, race/ethnicity, and place of origin), clinical data (treatment protocol, risk group, post-induction relapse, and death), and laboratory data (immunophenotype) were retrieved from physical medical records archived in the Medical and Statistical Care System (SAME) and from electronic medical records (iDoctor and Softlab systems) at HEMOAM. Race/ethnicity was defined by self-reported skin color or race, or by parental/legal guardian report for minors, according to the classification of the Brazilian Institute of Geography and Statistics (IBGE): White, Black, Brown (Mixed-race), Yellow (Asian), and Indigenous. In addition, relapse was defined as treatment failure occurring after completion of remission induction therapy (day 35), according to the treatment protocol. Death was assessed over a minimum follow-up period of five years after diagnosis.

### DNA extraction and assessment of nucleic acid purity

Genomic DNA was extracted using a commercial column-based method with the PureLink™ Genomic DNA Mini Kit (Invitrogen™), according to the manufacturer’s instructions. DNA concentration and purity were assessed by measuring absorbance ratios at 260/280 nm and 260/230 nm using a NanoDrop™ 2000/2000c spectrophotometer (Thermo Scientific™).

### SNV selection criteria and genotyping by quantitative PCR

Polymorphic variants were selected based on information retrieved from the Cancer Genome Anatomy Project and SNP500 databases. The selected variants included the TP53 missense variant *rs1042522* (c.215 C > G; p.Pro72Arg), located in exon 4, and the intronic variants *rs1642785* (C > G), located in intron 2, and *rs2909430* (C > T), located in intron 4. The selection criteria for single nucleotide variants (SNVs) included predicted functional relevance, minor allele frequency (MAF ≥ 3%), and previously reported associations with hematological malignancies, including leukemia. Genotyping of the selected variants was performed using quantitative real-time PCR (qPCR) with allele-specific TaqMan probes (Applied Biosystems) (Supplementary Table 1), following the protocol described by Alves et al. (2021) [11]. Amplification was carried out using the StepOnePlus™ Real-Time PCR System, and genotype assignment was performed using StepOne Software v2.3, based on amplification curve discrimination.

### Data analysis and statistical methods

Descriptive and statistical analyses were performed using GraphPad Prism version 10 (San Diego, CA, USA). Comparisons between groups were conducted using Fisher’s exact test, with results expressed as odds ratios (OR) and 95% confidence intervals (95% CI). Overall survival (OS) was defined as the time from diagnosis to death from any cause and was estimated using the Kaplan–Meier method, with differences between groups assessed by the log-rank test.

Hardy–Weinberg equilibrium (HWE) for all SNVs was evaluated using R software version 4.4.1 (www.r-project.org) with the *SNPassoc* package. The same package was used to assess associations between allele and genotype frequencies and susceptibility to ALL, relapse, and death under four genetic inheritance models: codominant, dominant, recessive, and overdominant. The best-fitting genetic model was selected based on the Akaike Information Criterion (AIC). P-values were adjusted for multiple comparisons using the Bonferroni correction, and statistical significance was defined as *p* < 0.025. Combined analyses (gene–gene interactions) of the SNVs were also performed using the *SNPassoc* package, considering *p* < 0.05 as statistically significant. Linkage disequilibrium (LD) was assessed using the *haplo.stats* package. D′ was used to estimate the strength of disequilibrium, and pairwise r² was used to measure the correlation between allele frequencies. The strength of correlation based on r² values was interpreted as strong (≥ 0.8), moderate (0.3 to < 0.8), or weak (< 0.3). Only haplotypes with a frequency greater than 5% were included in comparative analyses.

## Results

### Sociodemographic, clinical, and epidemiological profile of the study population

A total of 215 healthy individuals were included in the control group, with a median age of 29 years. The case group comprised 193 patients diagnosed with acute lymphoblastic leukemia (ALL), with a median age of 9 years. In both groups, males were predominant, accounting for 67% of the control group and 59% of the ALL group. Regarding racial distribution, 82% of patients with ALL self-identified as Brown (Mixed-race). All individuals in the control group (100%) resided in Manaus, while 71% of patients in the ALL group also reported residence in the capital city. Among 70 patients with available information, 56% reported no family history of cancer (Table 1).

**Table 1:** Sociodemographic, laboratory and clinical characteristics of the study population

Variables (completeness)	Controls *n* = 215	ALL *n* = 193
Age, n (%)	215 (100)	191 (98)
years, median [IQR]^a^	29 [4–65]	9 [0–69]
Sex	215 (100)	193 (100)
Male, n (%)	145 (67)	114 (59)
Female, n (%)	70 (33)	79 (41)
Ethnicity		120 (62)
White, n (%)	-	12 (10)
Admixed, n (%)	-	99 (82)
Other, n (%)	-	9 (8)
Immunophenotype		193 (100)
B-ALL, n (%)	-	176 (91)
T-ALL, n (%)	-	17 (9)
Residence		191 (98)
Manaus, n (%)	215 (100)	136 (71)
Interior of Amazonas, n (%)	-	53 (28)
Other state, n (%)	-	2 (1)
Risk Group		179 (92)
Low Risk, n (%)	-	58 (32)
High Risk, n (%)	-	121 (68)
Treatment protocol		179 (92)
GBTLI, n (%)	-	57 (32)
BFM, n (%)	-	90 (50)
GMALL, n (%)	-	22 (12)
HyperCVAD, n (%)	-	3 (2)
GMALL + HyperCVAD, n (%)	-	7 (4)
Relapse		145 (75)
Yes, n (%)	-	68 (47)
No, n (%)	-	77 (53)
Death		185 (96)
Yes, n (%)	-	54 (29)
No, n (%)	-	131 (71)
Family history of cancer		70 (36)
Yes, n (%)	-	31 (44)
No, n (%)	-	39 (56)

*IQR* Interquantile Range

Other: black 0 (0%), indigenous 8 (6.67%) and yellow 1 (0.83%). Other states: Pará 1 (0.52%) and Roraima 1 (0.52%). European Group Berlin-Frankfurt-Münster (BFM). Brazilian Group for the Treatment of Leukemia in Childhood (GBTLI). Group for Adult Acute Lymphoblastic Leukemia (GMALL). Ethnicity was defined by self-declaration of color or race or declaration by parents/guardians for minors, following the classification used by the Brazilian Institute of Geography and Statistics (IBGE): white, black, brown, yellow, and indigenous

With respect to immunophenotype, 91% of patients with ALL presented with B-cell ALL (B-ALL), whereas 9% had T-cell ALL (T-ALL). Additionally, 68% of patients were classified into the high-risk group. Most patients were treated according to the Berlin Frankfurt Münster (BFM) European Group protocol (50%), while 32% received treatment following the Brazilian Childhood Leukemia Treatment Group (GBTLI) protocol. During treatment, 53% of patients did not relapse after remission induction, and 71% were alive at the end of follow-up (Table 1).

### Allele frequencies and their association with prognosis in patients with acute lymphoblastic leukemia

Allele frequency analysis revealed a higher prevalence of the C allele in both study groups. However, comparative analysis showed that the C allele was more frequent in the case group than in the control group, whereas a higher proportion of the G allele was observed among healthy individuals (Supplementary Figs. 1 A and 1B).

Allelic association analysis demonstrated that the C allele of *rs1042522* was associated with an increased risk of developing ALL compared with the G allele (OR: 1.44; 95% CI: 1.06–1.96; *p* = 0.021) (Table 2). In addition, the allele C of *rs1042522* was associated with a higher risk of relapse (OR: 1.97; 95% CI: 1.14–3.32; *p* = 0.016) (Table 3) and death (OR: 2.15; 95% CI: 1.21–2.87; *p* = 0.009) (Table 4).

**Table 2:** Multivariate analysis adjusted for sex and age for the association of single nucleotide variants (SNVs) with the *risk* of acute lymphoblastic leukemia

Genetic models	Controls *n* = 215 (%)		ALL *n* = 193 (%)	OR (95% CI)	*p* value	AIC	OR (95% CI) adj	*p* value adj	AIC
rs1042522 C > G (HWE:1.0000)									
Codominant									
C/C		96 (44.7)	106 (54.9)						
C/G		95 (44.2)	75 (38.9)	0.71 (0.47–1.08)	0.056	564.7	0.75 (0.46–1.23)		443.7
G/G		24 (11.2)	12 (6.2)	0.45 (0.21–0.95)			0.34 (0.14–0.79)		
Dominant									
C/C		96 (44.7)	106 (54.9)						
C/G-G/G		119 (55.3)	87 (45.1)	0.66 (0.45–0.98)	***0.038***	564.1	0.65 (0.41–1.03)	0.067	445.1
Recessive									
C/C-C/G		191 (88.8)	181 (93.8)						
G/G		24 (11.2)	12 (6.2)	0.53 (0.26–1.09)	0.075	565.3	0.39 (0.17–0.88)	***0.019***	443.0
Overdominant									
C/C-G/G		120 (55.8)	118 (61.1)						
C/G		95 (44.2)	75 (38.9)	0.80 (0.54–1.19)	0.275	567.2	0.89 (0.56–1.42)	0.637	448.3
Alleles									
C		287 (66.74)	287 (74.35)	1.44 (1.06–1.96)	***0.021***				
G		143 (33.26)	99 (25.65)						
Log-Additive 0,1,2		215 (52.7)	193 (47.3)	0.69 (0.51–0.94)	***0.017***	562.7	0.65 (0.45–0.92)	***0.014***	442.6

*rs1642785* C > G (HWE: 0.5363)									
Codominant									
C/C		101 (47.0)	111 (57.5)						
C/G		95 (44.2)	73 (37.8)	0.70 (0.47–1.05)	0.054	564.6	0.65 (0.40–1.06)	***0.013***	441.9
G/G		19 (8.8)	9 (4.7)	0.43 (0.19–1.00)			0.28 (0.11–0.73)		
Dominant									
C/C		101 (47.0)	111 (57.5)						
C/G-G/G		114 (53.0)	82 (42.5)	0.65 (0.44–0.97)	***0.033***	563.9	0.58 (0.36–0.92)	***0.018***	443.0
Recessive									
C/C-C/G		196 (91.2)	184 (95.3)						
G/G		19 (8.8)	9 (4.7)	0.50 (0.22 − 1.14)	0.091	565.6	0.34 (0.14–0.86)	***0.018***	442.9
Overdominant									
C/C-G/G		120 (55.8)	120 (62.2)	0.77 (0.52–1.14)	0.191	566.7	0.77 (0.48–1.22)	0.259	447.2
C/G		95 (44.2)	73 (37.8)						
Alleles									
C		270 (69.07)	295 (76.42)	1.45 (1.06–1.97)	***0.022***				
G		116 (30.93)	91 (23.58)						
Log-Additive 0,1,2		215 (52.7)	193 (47.3)	0.68 (0.49–0.93)	***0.016***	562.7	0.59 (0.40–0.85)	***0.004***	440.4

Adjusted for sex and age (p-value adj, OR adj)

Significant associations with *p*<0.05 are represented in bold and italics

*OR* Odds Ratio, *p-value* *< 0.025*; 95% confidence interval, *AIC* Akaike Criterion Value, *HWE * Hardy-Weinberg equilibrium

**Table 3:** Association analysis of SNVs in the *TP53* gene with *relapse* in patients with acute lymphoblastic leukemia

Genetic models	No *n* = 77 (%)		Yes *n* = 68 (%)	OR (95% CI) adj	*p* value adj	AIC
rs1042522 C > G (HWE:1.0000)						
Codominant						
C/C	33 (43.4)		43 (63.2)			
C/G	36 (47.4)		23 (33.8)	0.52 (0.25–1.05)	0.073	196.5
G/G	7 (9.2)		2 (2.9)	0.26 (0.05–1.36)		
Dominant						
C/C	33 (43.4)		43 (63.2)			
C/G-G/G	43 (56.6)		25 (36.8)	0.48 (0.24–0.95)	***0.033***	195.2
Recessive						
C/C-C/G	69 (90.8)		66 (97.1)			
G/G	7 (9.2)		2 (2.9)	0.35 (0.07–1.78)	0.173	197.9
Overdominant						
C/C-G/G	40 (52.6)		45 (66.2)			
C/G	36 (47.4)		23 (33.8)	0.59 (0.30–1.18)	0.133	197.5
Alleles						
C	102 (67.11)		109 (80.15)	1.97 (1.14– 3.32)	***0.016***	
G	50 (32.89)		27 (19.85)			
Log-Additive 0,1,2	76 (52.8)		68 (47.2)	0.51 (0.29–0.92)	***0.022***	194.5

*rs1642785* C > G (HWE:0.5553)						
Codominant						
C/C	37 (48.7)		45 (66.2)		0.180	
C/G	35 (46.1)		20 (29.4)	0.51 (0.25–1.05)		198.3
G/G	4 (5.3)		3 (4.4)	0.80 (0.16–3.94)		
Dominant						
C/C	37 (48.7)		45 (66.2)	0.54 (0.27–1.07)	0.076	196.6
C/G-G/G	39 (51.3)		23 (33.8)			
Recessive						
C/C-C/G	72 (94.7)		65 (95.6)			
G/G	4 (5.3)		3 (4.4)	1.07 (0.22–5.11)	0.935	199.7
Overdominant						
C/C-G/G	41 (53.9)		48 (70.6)			
C/G	35 (46.1)		20 (29.4)	0.52 (0.26–1.05)	0.067	196.4
Alleles						
C	109 (71.71)		104 (80)	1.57 (0.91–2.73)	0.063	
G	43 (28.29)		26 (20)			
Log-Additive 0,1,2	76 (52.8)		68 (47.2)	0.64 (0.35–1.17)	0.141	197.6

Adjusted for sex and age (p-value adj, OR adj)

Significant associations with *p*<0.05 are represented in bold and italics

*OR* Odds Ratio, *p*-*value< 0.025* 95% confidence interval, *AIC* Akaike Criterion Value, *HWE* Hardy-Weinberg equilibrium

**Table 4:** Association analysis of SNVs in the *TP53* gene with *death* in patients with acute lymphoblastic leukemia

Genetic models	No *n* = 131 (%)	Yes *n* = 54 (%)	OR (95% CI) adj	*p* value adj	AIC
rs1042522 C > G (HWE:1.0000)					
Codominant					
C/C	63 (48.1)	37 (68.5)			
C/G	57 (43.5)	16 (29.6)	0.48 (0.24–0.95)	***0.018***	221.5
G/G	11 (8.4)	1 (1.9)	0.15 (0.02–1.25)		
Dominant					
C/C	63 (48.1)	37 (68.5)			
C/G-G/G	68 (51.9)	17 (31.5)	0.43 (0.22–0.83)	***0.010***	220.9
Recessive					
C/C-C/G	120 (91.6)	53 (98.1)			
G/G	11 (8.4)	1 (1.9)	0.21 (0.03–1.63)	0.067	224.1
Overdominant					
C/C-G/G	72 (56.5)	38 (70.4)	0.55 (0.28–1.08)	0.075	224.3
C/G	57 (43.5)	16 (29.6)			
Alleles					
C	183 (69.85)	90 (83.33)	2.15 (1.21–3.87)	***0.009***	
G	79 (30.15)	18 (16.67)			
Log-Additive 0,1,2	131 (70.8)	54 (29.2)	0.45 (0.25–0.81)	***0.005***	219.6

***rs1642785*** **C > G (HWE:0.5553)**					
Codominant					
C/C	68 (51.9)	37 (68.5)			
C/G	55 (42.0)	16 (29.6)	0.53 (0.27–1.06)	0.078	224.3
G/G	8 (6.1)	1 (1.9)	0.23 (0.03–1.91)		
Dominant					
C/C	68 (51.9)	37 (68.5)	0.50 (0.25–0.97)	***0.036***	223.0
C/G-G/G	63 (48.1)	17 (31.5)			
Recessive					
C/C-C/G	123 (93.9)	53 (98.1)			
G/G	8 (6.1)	1 (1.9)	0.29 (0.04–2.38)	0.182	225.6
Overdominant					
C/C-G/G	76 (58.0)	38 (70.4)	0.58 (0.29–1.15)	0.112	224.9
C/G	55 (42.0)	16 (29.6)			
Alleles					
C	191 (73.75)	90 (83.33)	1.78 (0.98–3.22)	0.058	
G	68 (26.25)	18 (16.67)			
Log-Additive 0,1,2	131 (70.8)	54 (29.2)	0.52 (0.29–0.94)	***0.024***	222.4

Adjusted for sex and age (p-value adj, OR adj)

Significant associations with *p*<0.05 are represented in bold and italics

*OR* Odds Ratio, *p*-*value* *< 0.025*; 95% confidence interval, *AIC* Akaike Criterion Value, *HWE* Hardy-Weinberg equilibrium

Similarly, the allele C of *rs1642785* was associated with increased susceptibility to ALL compared with the G allele (OR: 1.45; 95% CI: 1.06–1.97; *p* = 0.022). However, no statistically significant associations were observed between *rs1642785* and relapse (OR: 1.57; 95% CI: 0.91–2.73; *p* = 0.063) or death (OR: 1.78; 95% CI: 0.98–3.32; *p* = 0.058).

Assessment of Hardy-Weinberg equilibrium indicated that *rs2909430* was not in equilibrium and was monomorphic in the association analyses. Consequently, case-control comparative analysis for this SNV was not performed. Comparison of allele frequencies across populations showed similarity with the low frequency reported in East Asian populations (Supplementary Fig. 2). Furthermore, genotype frequencies of *rs1042522* and *rs1642785* in our study population were comparable to those observed in admixed American (AMR) populations from the 1000 Genomes Project (Supplementary Fig. 3). These comparisons reflect patterns of allele frequency distribution and should not be interpreted as direct estimates of genetic ancestry.

### Genotype frequencies and their association with prognosis in patients with acute lymphoblastic leukemia

The distribution of genotype frequencies for the polymorphic variants showed a similar pattern for both SNVs (*rs1042522* and *rs1642785*), with the CC, CG, and GG genotypes observed, respectively. Despite this shared distribution pattern, the CC genotype was more prevalent in cases than in controls, whereas the GG genotype was more frequent among controls for both *rs1042522* and *rs1642785* (Supplementary Figs. 1 C and 1D).

Adjusted analyses revealed that the *TP53* SNVs *rs1042522* (_adj_OR: 0.39; 95% CI: 0.17–0.88; *p* = 0.019) and *rs1642785* (_adj_OR: 0.65; 95% CI: 0.40–1.06; *p* = 0.013) were associated with a protective effect against the development of ALL after adjustment for age and sex (Table 2).

Table 3 presents the association analyses between genotypes and relapse following remission induction. In this cohort, *rs1042522* was significantly associated with protection against relapse (adjusted OR: 0.51; 95% CI: 0.29–0.92; adjusted *p* = 0.022), whereas *rs1642785* showed no statistically significant association (adjusted OR: 0.52; 95% CI: 0.26–1.05; adjusted *p* = 0.067).

Furthermore, both *rs1042522* (adjusted OR: 0.45; 95% CI: 0.25–0.81; adjusted *p* = 0.005) and *rs1642785* (adjusted OR: 0.52; 95% CI: 0.29–0.94; adjusted *p* = 0.024) were associated with a protective effect against death (Table 4).

### Interaction analysis of *rs1042522* and *rs1642785* on clinical outcomes

To evaluate interactions between the SNVs, a combined analysis of *rs1042522* and *rs1642785* was performed using a logistic regression model adjusted for age and sex. The results showed that, among the analyzed combinations and interactions, individuals heterozygous for *rs1042522* (CG) exhibited a protective effect against relapse compared with CC homozygotes (adjusted OR: 0.82; 95% CI: 0.25–0.98; *p* < 0.001).

No statistically significant associations were observed between genotype combinations and susceptibility to ALL or death. However, age emerged as a significant covariate influencing clinical outcomes. Increasing age was associated with an 8% reduction in the risk of ALL susceptibility per year (adjusted OR: 0.92; 95% CI: 0.90–0.93; *p* < 0.001), while it was also associated with a 4% increase in the risk of relapse following remission induction therapy (adjusted OR: 1.04; 95% CI: 1.00–1.07; *p* = 0.026), as shown in Supplementary Table 2.

### Impact of *TP53 rs1042522* and *rs1642785* on overall survival

**Fig. 1:**
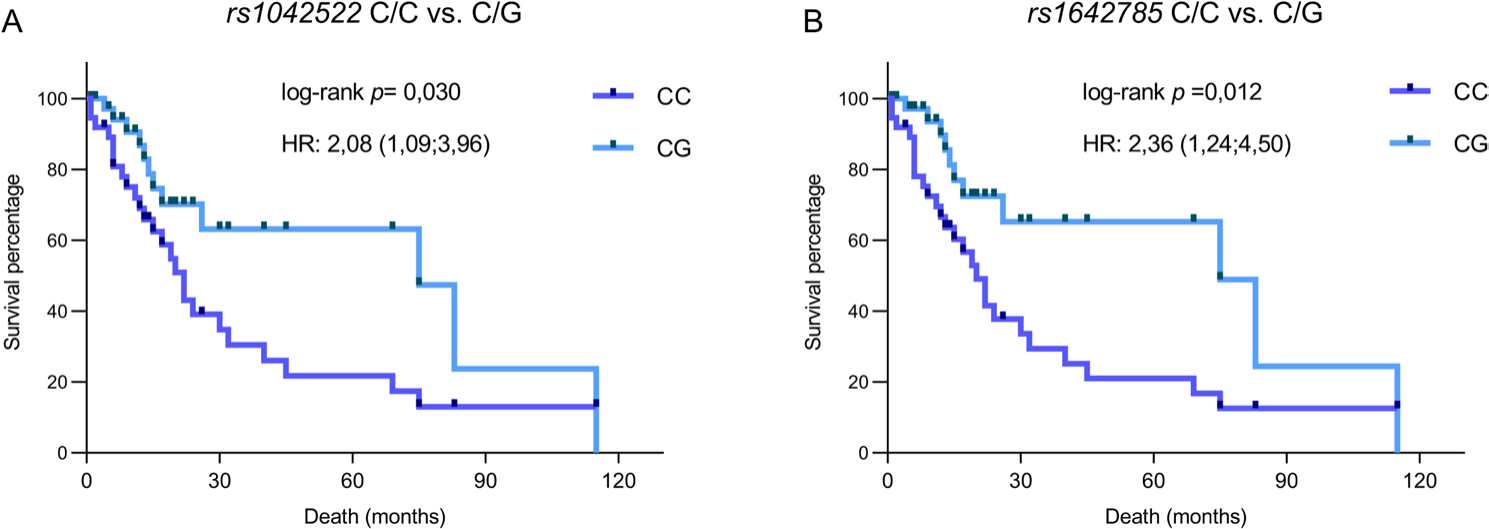
Overall survival of patients with acute lymphoblastic leukemia according to genotypes p-value (log-rank): < 0.05; HR: Hazard Ratio

Overall survival (OS) analysis according to *TP53* SNV genotypes revealed that specific *TP53* genotypes significantly influenced survival outcomes in patients with ALL. For *rs1042522*, individuals with the CC genotype were associated with a 2.08-fold higher mortality risk compared with patients harboring the CG genotype (*p* = 0.030; Fig. 1A), with a median survival of only 22 months (Table 5). In contrast, heterozygous CG individuals showed significantly prolonged overall survival, with a median of 75 months (Table 5).

**Table 5:** Overall survival analysis according to genotypes of the evaluated single nucleotide variants

Genetic models	ALL Deaths *n* = 44 (%)	Median Survival (months)	HR (95% CI)	Log-rank *p* value
rs1042522 C > G				
C/C	30 (68.2)	22.00	2.08 (1.09–3.96)	***0.030***
C/G	13 (29.5)	75.00		
G/G	1 (2.3)	-	-	-

***rs1642785*** **C > G**				
C/C	31 (70.5)	20.00	2.36 (1.24–4.50)	***0.012***
C/G	13 (29.5)	75.00		
G/G	0 (0)	-	-	-

Significant associations with *p*<0.05 are represented in bold and italics

*HR* Hazard Ratio (log-rank), *p value log-rank (Mantel-Cox) test* <0.05, *CI of Ratio 95%* Confidence interval

Similar findings were observed for *TP53 rs1642785*. Patients homozygous for the C allele (CC vs. CG) demonstrated a strong association with poorer survival (HR: 2.36; 95% CI: 1.24–4.50; *p* = 0.012), with a median survival of only 20 months (Table 5). Conversely, individuals with the heterozygous CG genotype of *rs1642785* exhibited a protective advantage (Fig. 1B).

### Haplotype analysis of *TP53 v*ariants

The polymorphic variants under investigation were found to be in moderate linkage disequilibrium (LD), with a D′ value of 0.893, indicating that they are frequently inherited together. The pairwise correlation coefficient (r²= 0.716) reflected a correlation between allele frequencies in the analysis of ALL susceptibility. Similar LD patterns were observed in analyses involving relapse and death, with D′ = 0.891 and r² = 0.711.

**Fig. 2:**
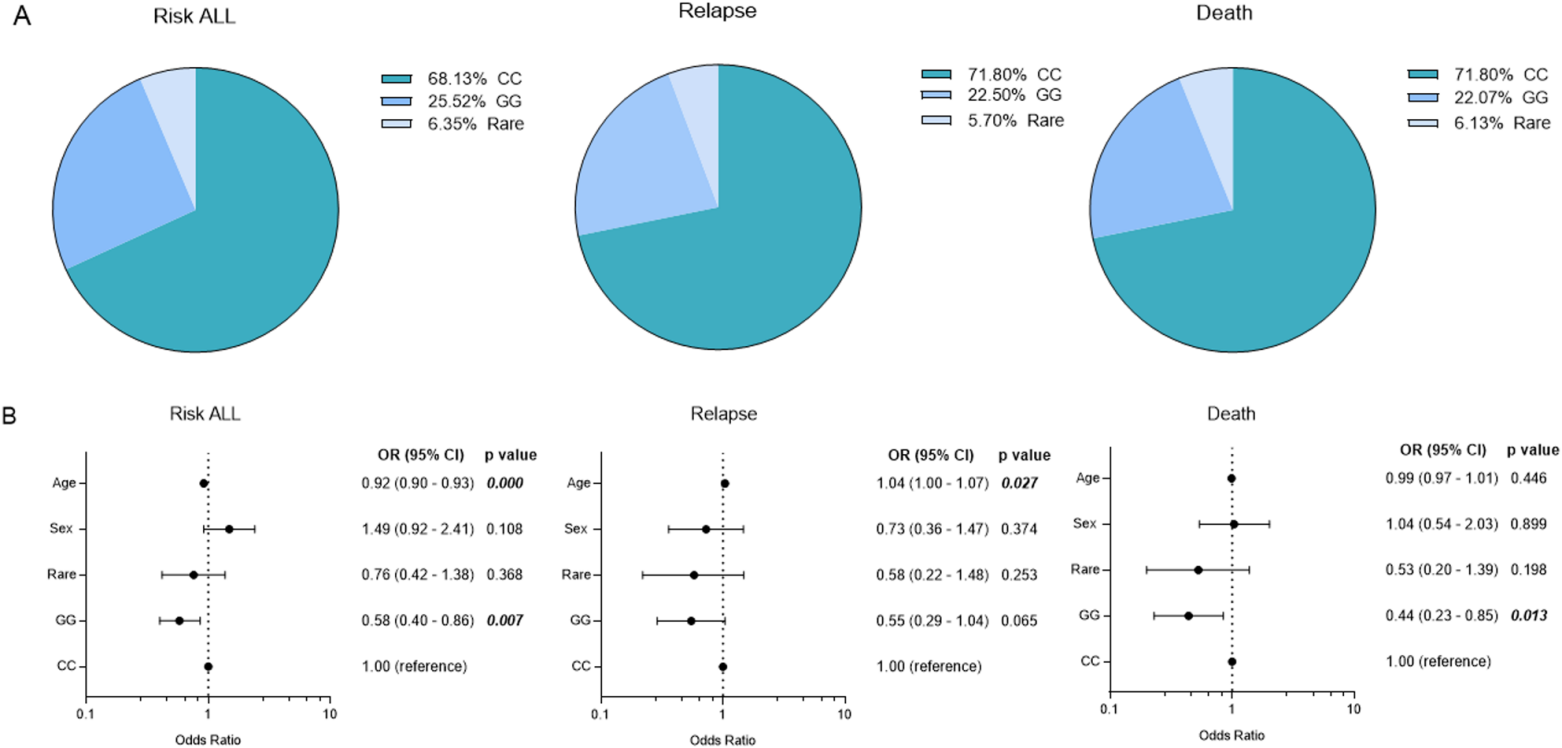
Frequency and association of the haplotypes identified in the studied population. OR Odds Ratio, 95% CI: 95% confidence interval

Four haplotypes were identified, of which two exhibited frequencies greater than 5%. The CC haplotype was the most prevalent in the studied population (Fig. 2A). Both common haplotypes showed significant associations with ALL. Individuals carrying the GG haplotype had a 42% lower risk of developing ALL compared with those carrying the CC haplotype (adjusted OR: 0.58; 95% CI: 0.40–0.86; *p* = 0.007), as well as a reduced risk of death (adjusted OR: 0.44; 95% CI: 0.23–0.85; *p* = 0.0138).

Age was identified as a significant covariate, with the risk of ALL decreasing by 8% per additional year of age (adjusted OR: 0.92; 95% CI: 0.90–0.93; *p* < 0.001), whereas the risk of relapse increased by 4% per year (adjusted OR: 1.04; 95% CI: 1.00–1.07; *p* = 0.0275).

The remaining two haplotypes showed frequencies below the predefined threshold and were therefore classified as rare haplotypes. These haplotypes did not demonstrate statistically significant associations with ALL susceptibility or clinical outcomes (adjusted OR: 0.76; 95% CI: 0.42–1.38; *p* = 0.368) (Fig. 2B).

## Discussion

The *TP53* gene plays a central role in maintaining genomic stability by regulating the expression of target genes involved in cell-cycle control mechanisms, thereby ensuring cellular homeostasis. However, *TP53* alterations are frequently observed across multiple human cancers [6]. Disruption of *TP53* may compromise the wild-type function of the p53 protein, contributing to tumor promotion and disease progression [7]. In acute lymphoblastic leukemia (ALL), alterations in the TP53/RB1 tumor suppressor pathway are commonly reported, particularly in high-risk patients [12]. Increasing evidence suggests that polymorphic variants in *TP53* are associated with susceptibility to ALL and may provide important insights into genetic factors influencing disease incidence and heterogeneity [5, 13].

In the present study, the clinical and sociodemographic characteristics of the study population were consistent with those reported in previous epidemiological investigations conducted in the same region [10]. Our findings demonstrated that the *TP53* the GG genotype of *rs1042522* genotype exerted a protective effect against ALL. These results are in agreement with the study by Skhoun et al. (2022), which reported that the CG and GG genotypes of *rs1042522* were associated with a protective effect against ALL in Moroccan children [14]. Nevertheless, the role of *rs1042522* appears to be context-dependent, with studies reporting heterogeneous findings ranging from no association to either protective or risk effects across different cancer types [15]. In Brazil, a single study conducted in a predominantly Caucasian population from the Southeast region reported an association between the allele C of *rs1042522* and increased susceptibility to ALL [16].

A meta-analysis reported that the CC genotype of *rs1042522* is correlated with an increased risk of ALL (OR = 1.73; 95% CI = 1.07–2.81) [17]. Although the specific effect of the CC genotype of *rs1042522* was not explicitly modeled as an isolated variable in our analysis, with findings suggest that this genotype is associated with increased susceptibility to ALL when compared with the CG and GG genotypes, given its higher prevalence in the case group. Similarly, Basabaeen et al. (2019) reported up to a tenfold increase in the risk of B-cell chronic lymphocytic leukemia (B-CLL) among individuals from the Sudanese population carrying the CC genotype compared with those harboring the GG genotype [18].

The *rs1642785* SNV also demonstrated a protective effect against ALL in our cohort. In patients with colorectal cancer diagnosed before the age of 57 years, the CG genotype of *rs1642785* has been reported to confer protection against disease development [19]. In contrast, the CC genotype has been associated with an increased risk of CLL (OR = 1.73; 95% CI = 1.01–2.95; *p* = 0.048) and with a higher frequency of somatic *TP53* mutations [9]. The CG genotype of *rs1642785* has also been identified in sporadic and familial cases of AML and ALL, exhibiting a similar degree of homozygosity to *rs1042522* in most cases [20].

To our knowledge, this is the first study to investigate the impact of *rs1642785* on ALL. Considering that ALL predominantly affects pediatric populations, findings from studies on CLL and AML may reflect genetic risk patterns primarily relevant to adult disease. Furthermore, combined analysis of the SNVs adjusted for age and sex demonstrated that increasing age was strongly associated with protection against ALL susceptibility, reinforcing the well-established epidemiological observation that ALL is more prevalent in children and has a lower incidence in adults [2, 10].

In the allele-based analysis, we observed that the C allele of both variants was associated with an increased risk of developing ALL. In patients with chronic lymphocytic leukemia (CLL), the C allele of *rs1042522* and *rs1642785* has been associated with higher expression of lipoprotein lipase (LPL) in leukemic B cells. Increased LPL expression may promote neoplastic progression by supplying metabolic substrates and creating a microenvironment favorable to cell survival and proliferation. In this context, Abramenko et al. (2021) suggested that these SNVs may act as genetic modifiers, influencing the development of CLL [21].

It has also been reported that the allele C of *rs1642785* affects mRNA stability, leading to reduced levels of p53 pre-mRNA and total *TP53* transcripts [22]. Reduced *TP53* transcription or impaired activation under conditions of cellular stress may compromise tumor suppressor function, thereby facilitating tumor development and progression [23]. Conversely, the allele G of *rs1642785* has been described as significantly more frequent in individuals with glioma than in control populations, suggesting that this variant may also confer increased risk in other oncological contexts [24].

Large genome-wide association studies (GWAS), including multi-ethnic analyses, have identified multiple susceptibility loci for childhood ALL but have not implicated common TP53 polymorphisms (MAF > 1%) as major risk factors outside rare syndromic contexts. In this context, our findings do not contradict GWAS evidence but rather suggest that the effect of common TP53 variants may vary across populations and may not be detectable in large, heterogeneous cohorts [25, 26].

The Brazilian Amazon population is highly admixed and remains underrepresented in genetic studies of childhood ALL. Differences in genetic background, including variation in linkage disequilibrium structure, may influence the observed associations. These findings reinforce the importance of investigating underrepresented populations, where context-dependent associations may not be captured in large-scale GWAS [27, 28].

To assess genetic variability, we compared allele frequencies across different populations using data from the 1000 Genomes Project. The frequencies of both SNVs in our cohort were similar to those reported in admixed American (AMR) populations. These comparisons reflect patterns of allele frequency distribution and should not be interpreted as direct estimates of genetic ancestry. The Brazilian population is known to be highly admixed, reflecting historical contributions from Indigenous peoples, Europeans, and Africans. Previous studies have described regional differences in genetic background across Brazil, including populations from the Northern region where the present study was conducted [29]. This genetic diversity may contribute to variability in allele frequencies and may partially account for differences observed across studies in literature. In this context, investigating genetic associations in admixed and underrepresented populations is important to better understand potential variability in susceptibility to ALL [11].

In addition, some authors have hypothesized that the frequency of the *rs1042522* variant may vary according to altitude, suggesting that Andean populations may have undergone genetic adaptation to environmental factors such as hypoxia, ultraviolet radiation, and extreme climatic conditions. Such adaptations may involve selective combinations of alleles and genotypes within the *TP53* pathway, further reinforcing the importance of considering genetic associations within a population and region-specific context [30].

To further investigate the prognostic impact of the studied variants in ALL, we evaluated their association with relapses, which remains a major therapeutic challenge. Combined SNV analysis revealed that individuals heterozygous for *rs1042522* (CG) had a lower risk of relapse compared with those carrying the CC genotype. In high-grade osteosarcoma, *rs1042522* has been associated with an increased risk of relapse [31], whereas data reported by Popek-Marciniec et al. (2023) showed no association between *rs1042522* and relapse risk in patients with multiple myeloma [32]. These findings highlight the context-dependent role of this variant across different malignancies.

Allele frequency analysis further demonstrated that the C allele was associated with an increased risk of relapse. It is well established that the C allele (Pro72) has a reduced ability to induce apoptosis in damaged cells, whereas the G allele (Arg72) is more effective due to its greater mitochondrial localization and enhanced transcription of several *TP53*-regulated pro-apoptotic genes. In contrast, the C allele is more efficient in promoting cell-cycle arrest, favoring DNA damage repair [33]. This functional divergence may help explain our findings, as individuals carrying the C allele may exhibit increased resistance to chemotherapy in this context.

Notably, inactivation of the allele C of *rs1042522* has been reported to influence the progression of BCR::ABL1-positive ALL. In approximately 12% of relapse cases, loss of heterozygosity of this allele was observed in leukemic cells, suggesting functional inactivation that may contribute to treatment resistance [34].

The *rs1642785* SNV exhibited a protective effect with respect to relapse. When evaluating the outcome of death, the log-additive model showed that both SNVs exerted a significant protective effect. However, the allele C of *rs1042522* was associated with an increased risk for this outcome. In multiple myeloma, *rs1042522* has been reported to have no significant impact on mortality [32]. Similarly, a large cohort study reported no significant association between *rs1042522* and cancer-related mortality [35]. Overall, data regarding the specific roles of these SNVs in relapse and death remain limited, particularly in hematological malignancies. To our knowledge, this is the first study to associate *rs1642785* with these clinical outcomes in ALL.

Overall survival analysis of both SNVs revealed that the CG genotype was associated with longer survival than the CC genotype in patients with ALL. Our combined analyses further reinforced the protective role of the heterozygous CG genotype, corroborating the findings observed across different analytical approaches. Skhoun et al. (2022) also suggested that the heterozygous genotype confers a survival advantage in patients with ALL [14]. In addition, the CG and GG genotypes have been associated with improved OS in glioblastoma [36]. Regarding *rs1642785*, a study in osteosarcoma similarly reported a protective effect, with associations to improved event-free survival and OS [31].

OS analysis also associated the CC genotype of both SNVs with poorer survival compared with the CG genotype. Indeed, *rs1042522* has previously been associated with reduced OS (*p* = 0.004), with a mean survival of only 12 months reported for patients with plasma cell myeloma carrying the CC genotype [37]. In acute myeloid leukemia (AML), genotype distributions did not show a significant impact on OS; however, *rs1042522* has been reported to reduce apoptotic potential, thereby increasing leukemia risk and being associated with poorer OS and higher relapse rates [8]. In addition, *rs1642785* has been shown to influence survival outcomes in patients with osteosarcoma [38].

Given the close genomic proximity of the studied SNVs, linkage disequilibrium analysis enabled haplotype-based evaluation, revealing that inherited allele combinations, particularly the GG haplotype, were associated with protection against ALL and death when compared with the CC haplotype. Preliminary evidence has suggested co-segregation of *rs1042522* and *rs1642785*, supporting the presence of linkage disequilibrium between these variants [20]. Age also emerged as an important variable in haplotype analyses, acting as an increasing risk factor for relapse with each additional year. In this regard, the literature consistently reports that older individuals with ALL tend to respond less favorably to therapy due to differences in tumor biology when compared with younger patients [39].

Despite the strengths of this study, several limitations should be acknowledged. The relatively modest sample size, compared with large-scale polymorphism studies, may have limited the statistical power of some analyses, particularly for less frequent genotype combinations. In addition, overall survival analyses were based on a limited number of events, as complete information on the date of death was available for only 44 patients. The low frequency of certain genotypes, particularly the GG genotype (Table 5), further reduced the ability to detect genotype-specific survival differences.

The analyzed variants may also be co-inherited as haplotypes, which could contribute to overlapping survival patterns. Moreover, functional and gene expression data were not available, limiting direct inference regarding the biological mechanisms underlying the observed associations.

Another limitation is the absence of direct ancestry estimation using ancestry informative markers (AIMs) or genome-wide data. Although cases and controls were recruited from the same healthcare setting and geographic region, which likely reduces major differences in population background, residual population stratification cannot be completely excluded. Therefore, the observed associations should be interpreted with caution and validated in future studies incorporating direct ancestry inference. Larger and multi-center studies, as well as functional investigations, will be essential to confirm and expand upon these findings.

## Conclusion

In conclusion, this study suggests that the *TP53* variants *rs1042522* and *rs1642785* are associated with susceptibility and clinical outcomes in patients with ALL from the Brazilian Amazon. Our findings indicate that these variants may influence the risk of disease development, relapse, and overall survival, with a potential protective effect observed for specific heterozygous genotypes and the GG haplotype. These results support the potential role of germline *TP53* variation as a prognostic biomarker in ALL, particularly in underrepresented and admixed populations. Furthermore, our findings highlight the importance of regional genetic studies and warrant further investigation in larger cohorts and functional analyses to better define the clinical utility of these variants in risk stratification and personalized approaches.

## Supplementary Information

Supplementary Material 1.

Supplementary Material 2.

Supplementary Material 3.

Supplementary Material 4.

Supplementary Material 5.
