# Supplementary material for: Association of TP53 polymorphic variants rs1042522 and rs1642785 with susceptibility and prognosis of acute lymphoblastic leukemia in a Brazilian Amazon population
Source: BMC Med Genomics. 2026 Apr 14;19:89. doi: 10.1186/s12920-026-02371-0 (PMC13191962; doi:10.1186/s12920-026-02371-0)
Supplement: Supplementary file 1 — Supplementary Material 1. [file 12920_2026_2371_MOESM1_ESM.docx]

**Supplementary Table 1.** Gene, SNVs, type, nucleotide substitution, chromosomal location, sequence of probes used in the experiment (VIC/FAM) and assay ID.

| **Gene** | **SNVs** | **Type** | **Substitution** | **Location** | **5’- [VIC/FAM] -3’** | **Assay ID** |
| --- | --- | --- | --- | --- | --- | --- |
|  | *rs1042522* | Missense | C>G | Chr17:7676154 | AGGAGCTGCTGGTGCAGGGGCCACG**[C/G]**GGGGAGCAGCCTCTGGCATTCTGGG | C___2403545_10 |
| ***TP53*** | *rs1642785* | Intron | C>G | Chr17:7676483 | TCTGCTAGGGGGCTGGGGTTGGGGT**[C/G]**GGGGGTGGTGGGCCTGCCCTTCCAAT | C___2880090_10 |
|  | *rs2909430* | Intron | C>T | Chr17:7675327 | GACGGCAGCAAAGAAACAAACATG**[C/T]**GTAAGCACCTCCTGCAACCCACTAG | C__26603915_10 |
