# Supplementary material for: Association of TP53 polymorphic variants rs1042522 and rs1642785 with susceptibility and prognosis of acute lymphoblastic leukemia in a Brazilian Amazon population
Source: BMC Med Genomics. 2026 Apr 14;19:89. doi: 10.1186/s12920-026-02371-0 (PMC13191962; doi:10.1186/s12920-026-02371-0)
Supplement: Supplementary file 2 — Supplementary Material 2. [file 12920_2026_2371_MOESM2_ESM.docx]

**Supplementary Table 2.** Combined analysis of the SNVs under study.

| **Variable - LLA risk** | **OR (95% CI)** | **OR (IC 95%) _Adj_** | ***p* value**  **(Wald)** | ***p* value**  **(LR)** |
| --- | --- | --- | --- | --- |
| ***rs1042522* C/G** | 0.73 (0.48 – 1.1) | 1.21 (0.36 – 4.09) | 0.758 | 1 |
| ***rs1042522* G/G** | 0.46 (0.22 – 0.97) | 0.69 (0.09 – 5.49) | 0.724 | 1 |
| ***rs1642785* C/G** | 0.71 (0.47 – 1.07) | 0.21 (0.02 – 2.35) | 0.207 | 1 |
| ***rs1642785* G/G** | 0.44 (0.19 – 1.01) | 0.75 (0.07 – 7.71) | 0.810 | 1 |
| **Sex** | 1.43 (0.95 – 2.15) | 1.46 (0.89 – 2.38) | 0.132 | 0.131 |
| **Age** | 0.92 (0.9 – 0.93) | 0.92 (0.9 – 0.93) | ***<0.001*** | ***<0.001*** |
| ***rs1042522* C/G:** ***rs1642785*C/G** | **-** | 2.71 (0.18 – 39.9) | 0.469 | 0.71 |
| ***rs1042522*G/G: *rs1642785*C/G** | **-** | 2.87 (0.09 – 96.68) | 0.556 | 0.71 |
| ***rs1042522*C/G: *rs1642785*G/G** | **-** | 0 (0 – ∞) | 0.987 | 0.71 |
| ***rs1042522*G/G: *rs1642785*G/G** | **-** | 0.48 (0.02 – 12.5) | 0.657 | 0.71 |
| **Variable - Relapse** | **OR (95% CI)** | **OR (IC 95%) _Adj_** | ***p* value**  **(Wald)** | ***p* value**  **(LR)** |
| ***rs1042522* C/G** | 0.49 (0.25 – 0.98) | 0.82 (0.25 – 0.98) | 0.82 | ***<0.001*** |
| ***rs1042522* G/G** | 0.22 (0.04 – 1.13) | 0 (0 – ∞) | 0.992 | ***<0.001*** |
| ***rs1642785* C/G** | 0.47 (0.23 – 0.95) | 1.87 (0 – ∞) | 1 | 1 |
| ***rs1642785* G/G** | 0.62 (0.13 – 2.93) | 1.22 (0.07 – 21.06) | 0.892 | 1 |
| **Sex** | 0.81 (0.41 – 1.57) | 0.7 (0.34 – 1.44) | 0.327 | 0.325 |
| **Age** | 1.04 (1.01 – 1.07) | 1.04 (1 – 1.07) | ***0.026*** | ***0.021*** |
| ***rs1042522* C/G:** ***rs1642785*C/G** | **-** | **-** | 1 | 0.477 |
| ***rs1042522*G/G: *rs1642785G*/G** | **-** | **-** | 0.992 | 0.477 |
| **Variable - Death** | **OR (95% CI)** | **OR (IC 95%) _Adj_** | ***p* value**  **(Wald)** | ***p* value**  **(LR)** |
| ***rs1042522* C/G** | 0.48 (0.24 – 0.95) | 0.29 (0.03 – 2.54) | 0.261 | 1 |
| ***rs1042522* G/G** | 0.15 (0.02 – 1.25) | 0 (0 – ∞) | 0.992 | 1 |
| ***rs1642785* C/G** | 0.53 (0.27 – 1.06) | 0 (0 – ∞) | 0.995 | 1 |
| ***rs1642785* G/G** | 0.23 (0.03 – 1.91) | 0.76 (0.06 – 8.81) | 0.823 | 1 |
| **Sex** | 0.98 (0.51 – 1.87) | 1.03 (0.52 – 2.04) | 0.929 | 0.929 |
| **Age** | 0.99 (0.97 – 1.01) | 0.99 (0.96 – 1.01) | 0.536 | 0.531 |
| ***rs1042522* C/G:** ***rs1642785*C/G** | **-** | 0 (0 – ∞) | 0.994 | 0.432 |
| ***rs1042522*G/G: *rs1642785*C/G** | **-** | 0 (0 – ∞) | 0.991 | 0.432 |
| ***rs1042522*G/G: *rs1642785*G/G** | **-** | 1.31 (0 – ∞) | 1 | 0.432 |

OR: Odds Ratio; adjusted for sex and age (OR adj); p-value: < 0.05; 95% confidence interval; 0: There were no events observed for this combination.
