# Supplementary figures and images for: Association of TP53 polymorphic variants rs1042522 and rs1642785 with susceptibility and prognosis of acute lymphoblastic leukemia in a Brazilian Amazon population
Source: BMC Med Genomics. 2026 Apr 14;19:89. doi: 10.1186/s12920-026-02371-0 (PMC13191962; doi:10.1186/s12920-026-02371-0)

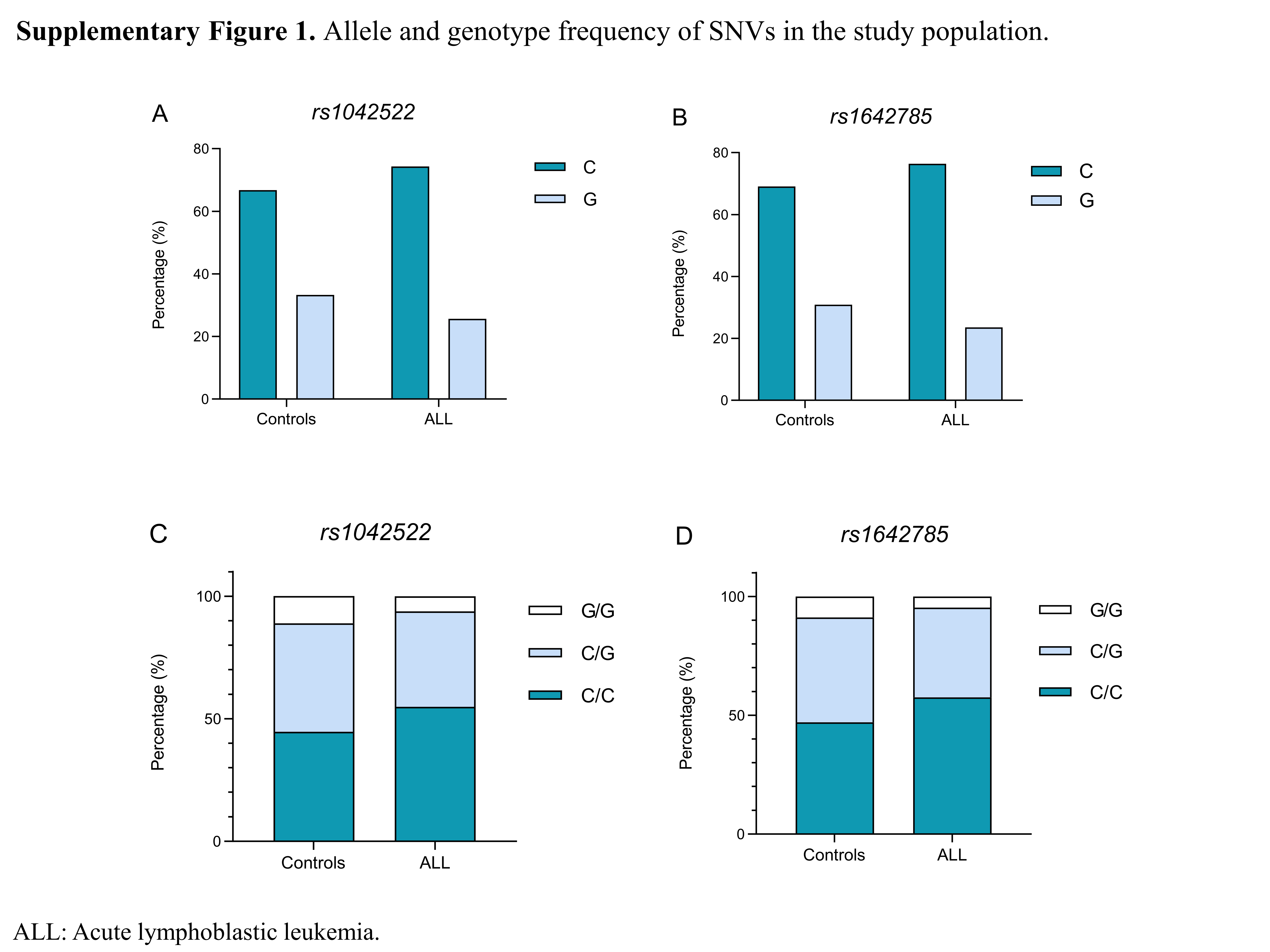

Supplement: Supplementary file 3 — Supplementary Material 3. [file 12920_2026_2371_MOESM3_ESM.tiff]

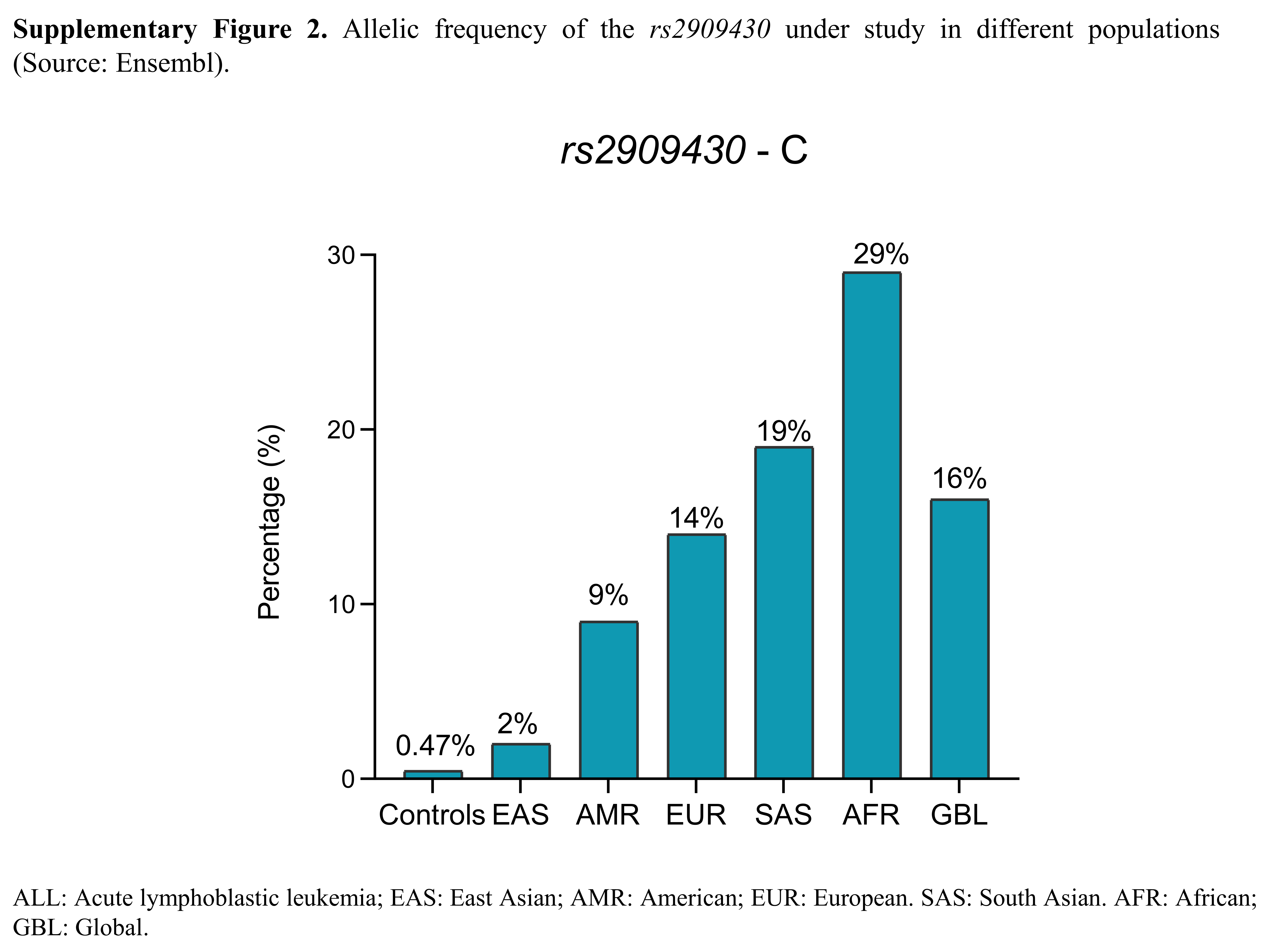

Supplement: Supplementary file 4 — Supplementary Material 4. [file 12920_2026_2371_MOESM4_ESM.tiff]

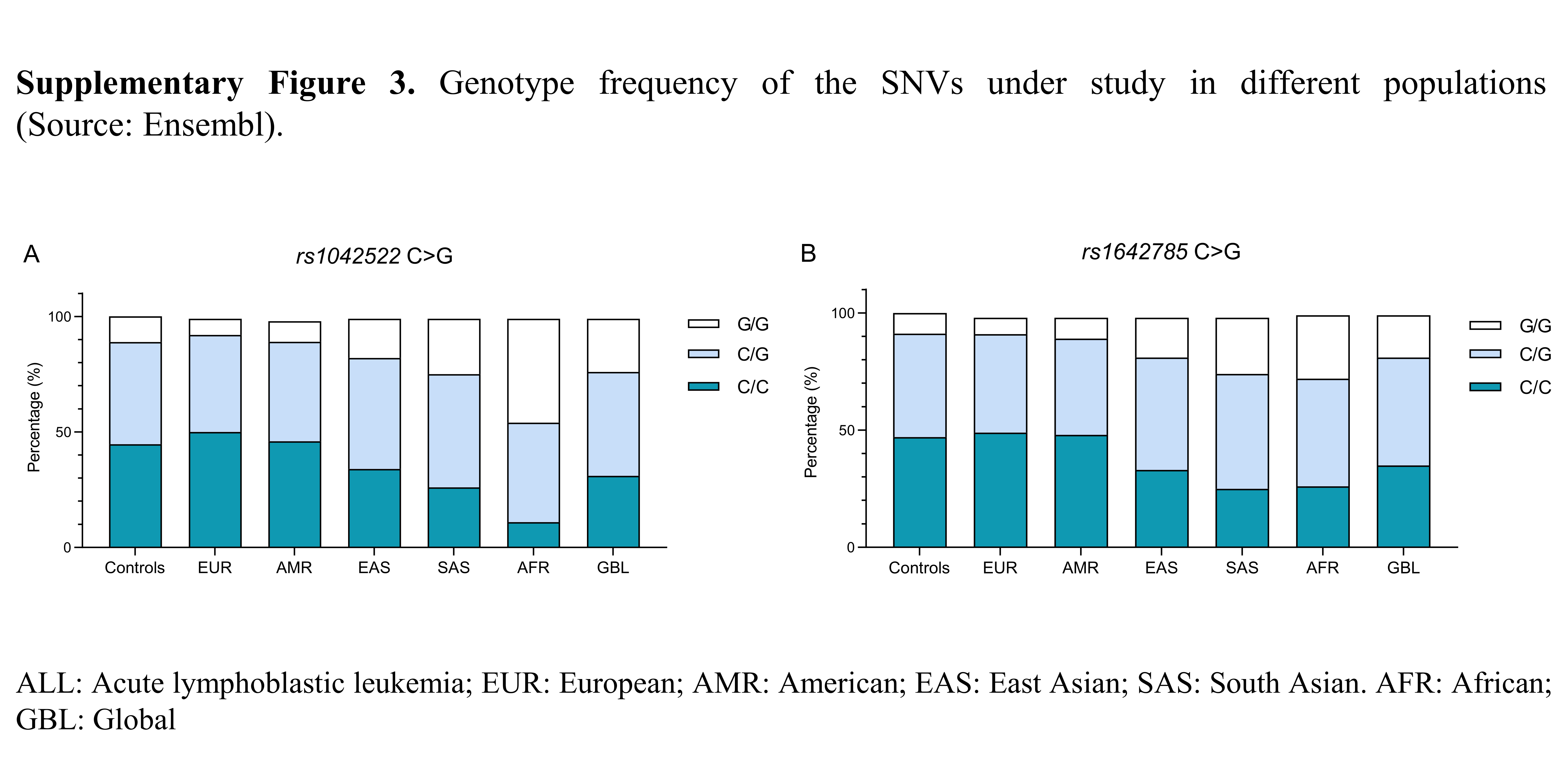

Supplement: Supplementary file 5 — Supplementary Material 5. [file 12920_2026_2371_MOESM5_ESM.tiff]
